# Canalicular adenoma with unicystic morphology. A rare entity

**DOI:** 10.4317/jced.57646

**Published:** 2021-01-01

**Authors:** Efstathios Pettas, Vasileios-Ionas Theofilou, Maria Georgaki, Argyrios Daskalopoulos, Demos Kalyvas, Andreas C. Lazaris, Rania H. Younis, Nikolaos G. Nikitakis

**Affiliations:** 1 DDS. Department of Oral Medicine and Pathology, School of Dentistry, National and Kapodistrian University of Athens (NKUA), Athens, Greece; 2 DDS. Department of Oral Medicine and Pathology, School of Dentistry, National and Kapodistrian University of Athens (NKUA), Athens, Greece. Department of Oncology and Diagnostic Sciences, School of Dentistry, University of Maryland Baltimore (UMB), Baltimore, Maryland, USA; 3 DDS, MSc. Department of Oral Medicine and Pathology, School of Dentistry, National and Kapodistrian University of Athens (NKUA), Athens, Greece; 4 DDS, MSc, PhD. Department of Oral Medicine and Pathology, School of Dentistry, National and Kapodistrian University of Athens (NKUA), Athens, Greece; 5 DDS, PhD. Department of Oral and Maxillofacial Surgery, School of Dentistry, National and Kapodistrian University of Athens, Athens, Greece; 6 MD, PhD. 1st Department of Pathology, School of Medicine, National and Kapodistrian University of Athens (NKUA), Athens, Greece; 7 BDS, MDS, PhD. Department of Oncology and Diagnostic Sciences, School of Dentistry, University of Maryland Baltimore (UMB), Baltimor, Maryland, USA. The Marlene and Stewart Greenebaum Cancer Center, University of Maryland Baltimore (UMB), Baltimore, Maryland, USA. Department of Oral Pathology, Faculty of Dentistry, Alexandria University, Alexandria, Egypt; 8 MD, DDS, PhD. Department of Oral Medicine and Pathology, School of Dentistry, National and Kapodistrian University of Athens (NKUA), Athens, Greece

## Abstract

**Background:**

Canalicular adenoma (CA) is a benign salivary gland tumor (SGT) almost exclusively affecting the minor salivary glands, predominantly of the upper lip, and exhibiting characteristic histopathologic features. As observed in several other SGTs, a commonly encountered finding is the presence of prominent cystic morphology. Even though a multicystic appearance is usually noticed, solitary cystic CAs may rarely occur.

**Case Report:**

Two female patients (74 and 78 years old respectively) presented for the evaluation of submucosal asymptomatic masses of the oral cavity. In the 1st case a solitary nodule was noticed in the upper lip, while the 2nd patient exhibited two symmetrical lesions of the buccal mucosae. All three excised specimens displayed cystic morphology upon gross examination. Histopathologically, a solitary cystic formation lined by monomorphic cuboidal or basaloid cells arranged in solid or trabecular patterns was observed in the 1st case. With a differential diagnosis of CA vs basal cell adenoma immunohistochemical examination was performed. Positivity for S-100, CK7 and CD117 (c-kit) and negative reaction for GFAP, p63 and SMA rendered the diagnosis of CA. In the 2nd case both lesions displayed well-circumscribed proliferations by monotonous cuboidal or columnar cells arranged in single cords and occasionally forming beading patterns, while central solitary areas of marked cystic degeneration were noticed. Diagnosis of multifocal unicystic CA was disclosed.

**Discussion:**

To our knowledge, only 11 additional cases of unicystic CA have been reported in the English-language literature. Although the exact clinical significance of unicystic morphology in CA is unknown, a tendency for occurrence within the context of multifocal tumors has been detected.

** Key words:**Canalicular adenoma, monomorphic adenoma, unicystic morphology, multifocal tumors, minor salivary glands.

## Introduction

Canalicular adenoma (CA) is a benign salivary gland tumor (SGT) that primarily affects the upper lip of patients in their 7th decade of life with a female predominance (1,2). Its clinical manifestations are usually non-pathognomonic, most commonly presenting as a solitary, slowly growing, freely movable asymptomatic nodule of the upper lip (2). However, peculiar clinicopathologic findings may occasionally be observed in these cases causing diagnostic dilemmas. Such examples include the occurrence of prominent cystic morphology which can be reminiscent of other neoplastic or reactive lesions of the oral cavity as well as the rarely encountered phenomenon of multifocal and/or relapsing involvement (3).

Besides the clinical similarities with diverse conditions of the oral mucosa, occasional confusion between different entities in the spectrum of monomorphic adenomas - especially basal cell adenoma (BCA) - could be attributed to the overlap observed regarding their histopathologic features. Even up to now, differentiating CA from BCA may be challenging, as a result of histopathologic similarities and may require immunohistochemical evaluation in certain instances. The main histopathologic finding in CA is the proliferation of benign cuboidal or columnar cells with monomorphic features, forming anastomosing cords, tubules or beading structures in an inconspicuous myxoid and vascular stroma (1,4). The tumor is mostly encapsulated with peripheral bosselation or lobulation (1).

Even though these features are almost entirely present in every single case, variations or uncommon histopathologic findings may be encountered (1). The presence of squamous balls/morules, noticeable hemorrhage or calcifications characterized as psammoma bodies have occasionally been detected (1). Other less characteristic histopathologic features may also be noticed in the stroma of these tumors, such as the presence of sclerotic changes/collagenization and lipofuscin/laden histiocytes, with the latter also being discerned in the luminal area (1). Additionally, CA may exhibit multifocality, which may result in incomplete removal with positive tumor margins, or a multinodular architecture (1). Mitoses (1) or in rare instances necrosis (5) have been seen in CA and could cause confusion with malignant salivary gland neoplasms. Finally, unique findings such as mucinous or oncocytic metaplasia, as well as tyrosine crystals, have also been described (1).

Cystic degeneration, a common finding in SGTs, is also a frequently encountered feature that characterizes the histopathologic phenotype of CA (1,6). This phenomenon, which has been correlated with distinct clinical features as well as the biologic behavior of certain neoplasms of the salivary glands, exhibits varying degrees of expression in CA (1). More specifically, even though these degenerative processes are usually pathologically translated into the formation of multiple microcystic spaces (1), cases with distinctive cystic morphology leading to the formation of a solitary cystic space have rarely been reported (7). This prominent cystic morphology exhibits a clinical significance as the true neoplastic behavior of the lesion may be masqueraded. Additionally, it may predispose towards specific clinical and/or prognostic characteristics that have not been highlighted to this date. Herein, we report two cases of unicystic CA and review the English-language literature. Interestingly, one of our cases involved a patient with multifocal lesions, which is in accordance with similar previously reported cases of unicystic features in multifocal CAs.

## Case Report

-Case 1

**Figure 1:**
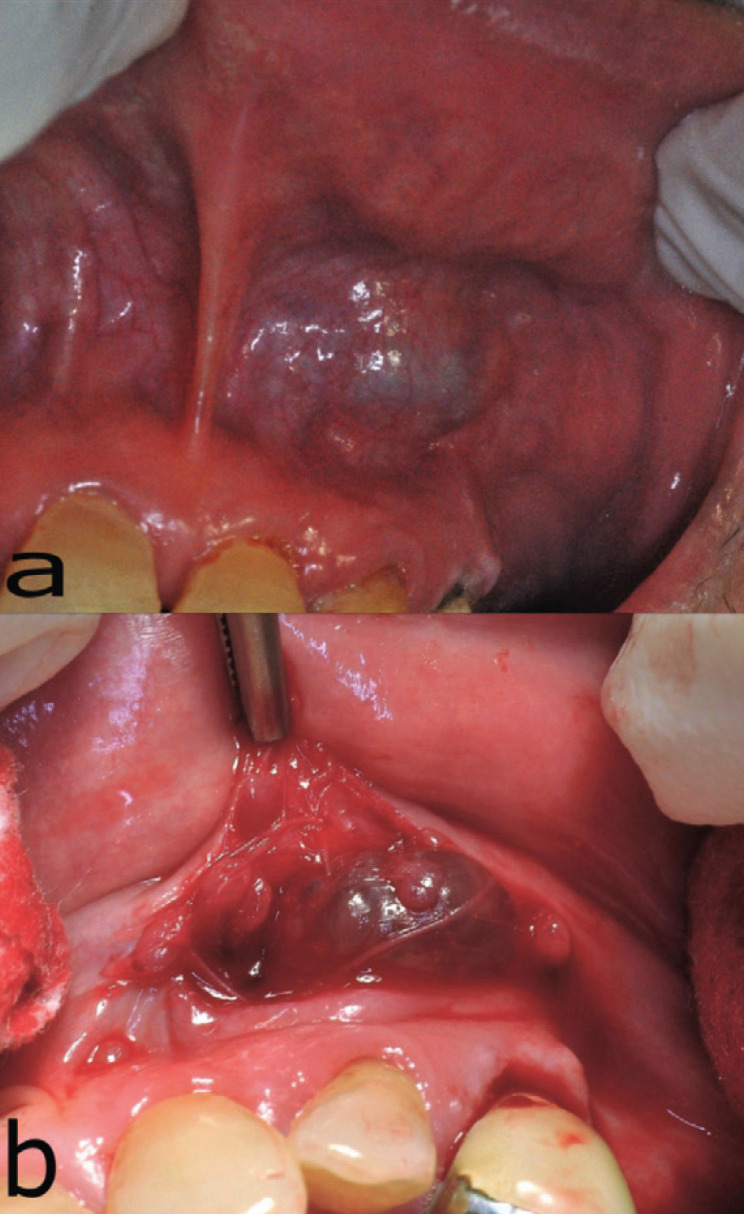
Patient 1. Clinical aspect of the lesion. a. Submucosal nodule involving the upper lip covered by intact mucosa with bluish color. b. Intraoperatively, a well circumscribed lesion of cystic morphology was obvious.

A 74 year-old Caucasian female of unremarkable medical history presented for the evaluation of a painless slowly-growing swelling on her upper lip of several months duration. She did not report any other symptoms in the oral cavity and could not correlate the development of the lesion with any possible causal factor. Clinical examination revealed a bluish fluctuant submucosal mass covered by intact mucosa located on the left side of the upper lip towards the mucolabial fold (Fig. 1a). The rest of the oral mucosa was within normal limits. With a provisional diagnosis of benign SGT, excisional biopsy of the nodule under local anesthesia was performed. Intraoperatively, a well circumscribed lesion of cystic morphology was noticed (Fig. 1b).

**Figure 2:**
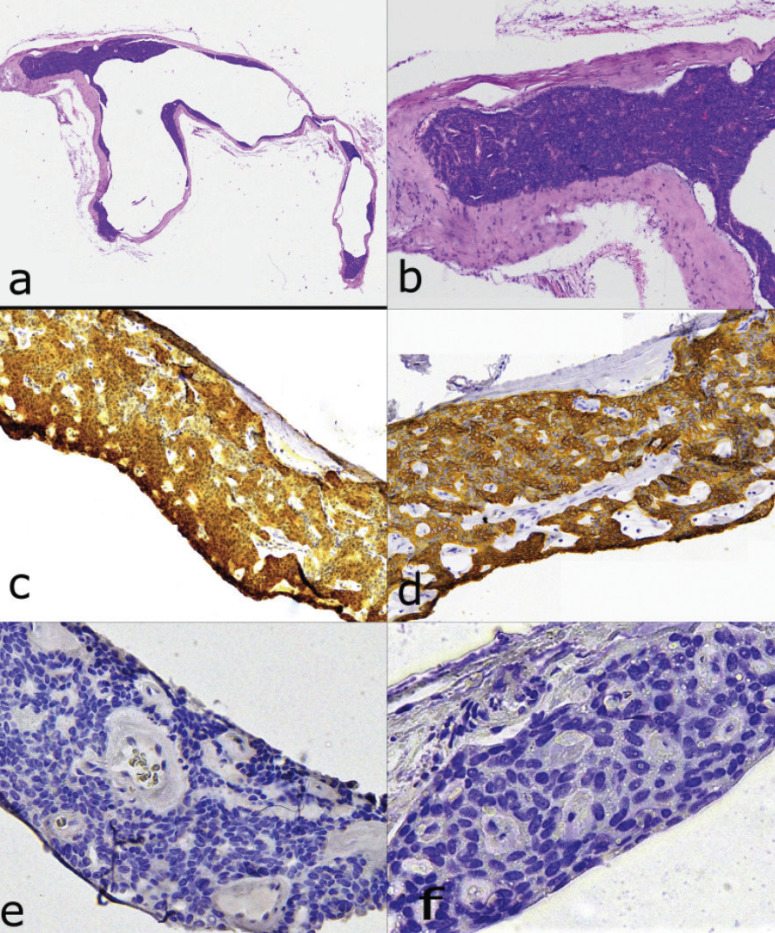
Patient 1. Histopathologic features of the lesion. a. Well circumscribed enlarged solitary cystic cavity lined by monotonous basaloid cells b. Cuboidal, basophilic tumor cells with absence of nuclear atypia and mitotic activity forming solid structures in a minimum vascularized stroma. *Hematoxylin and eosin stain, initial magnification 200x*. Immunohistochemical examination showed positivity of the neoplastic cells for S-100 (c), CK7 (d) and CD117 (c-kit) as well as negative reaction for GFAP (e), p63 (f) and SMA. *Initial magnification 400x*.

On gross examination, the lesion measured 0.7x0.6x0.5 cm; hemisecting the specimen revealed a central cystic space. Histopathologic examination showed a well-circumscribed, encapsulated unicystic lesion lined by aggregates of basophilic cells, which formed variably sized thickenings partially protruding into the cystic lumen (Fig. 2a). The neoplastic cell population was monotonous, composed of cuboidal or basaloid cells, presenting ovoid dark-stained nuclei and scant eosinophilic cytoplasm. They were arranged in solid or trabecular formations with minimal amounts of surrounding paucicellular fibrous stroma, while typical beading patterns were not observed. Mitotic Figures, pleomorphism or stromal necrosis were not noticed (Fig. 2b).

The histopathologic features raised a differential diagnosis between CA and BCA. While the demographic features, site of involvement and prominent cystic degenerative changes were more in keeping with CA, BCA was also considered due to the absence of typical beading pattern as well as the basaloid morphology of the cells. Immunohistochemical examination was performed showing diffuse positivity of the neoplastic cells for S-100 (Fig. 2c), CK7 (Fig. 2d) and CD117 (c-kit); on the other hand, the tumor cells were negative for GFAP (Fig. 2e), p63 (Fig. 2f) and SMA. Based on these combined findings, a diagnosis of unicystic CA was made.

-Case 2

**Figure 3:**
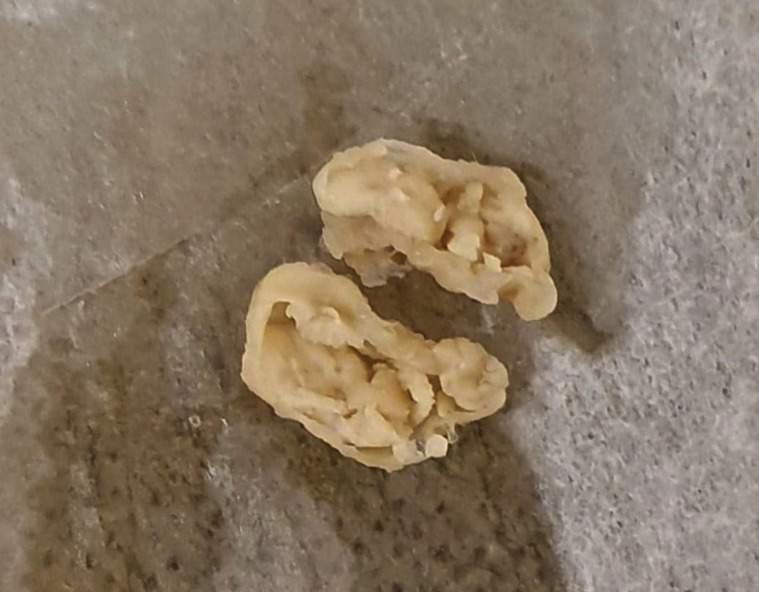
Patient 2. Gross examination of the right buccal nodule. A tan soft-tissue specimen exhibiting prominent cystic morphology (after hemi-section) could be observed.

A 78 year-old white female of non-significant medical history presented with bilateral movable soft tissue masses involving the buccal mucosae. The remaining oral mucosa was within normal limits. With provisional diagnosis of reactive pathoses of the minor salivary glands, excisional biopsy of both nodules was performed. Upon gross examination, two soft tissue specimens with a maximum diameter of 1.0 cm for the right lesion and 0.7 cm for the left lesion in addition to a prominent cystic morphology, were observed: (Fig. 3).

**Figure 4:**
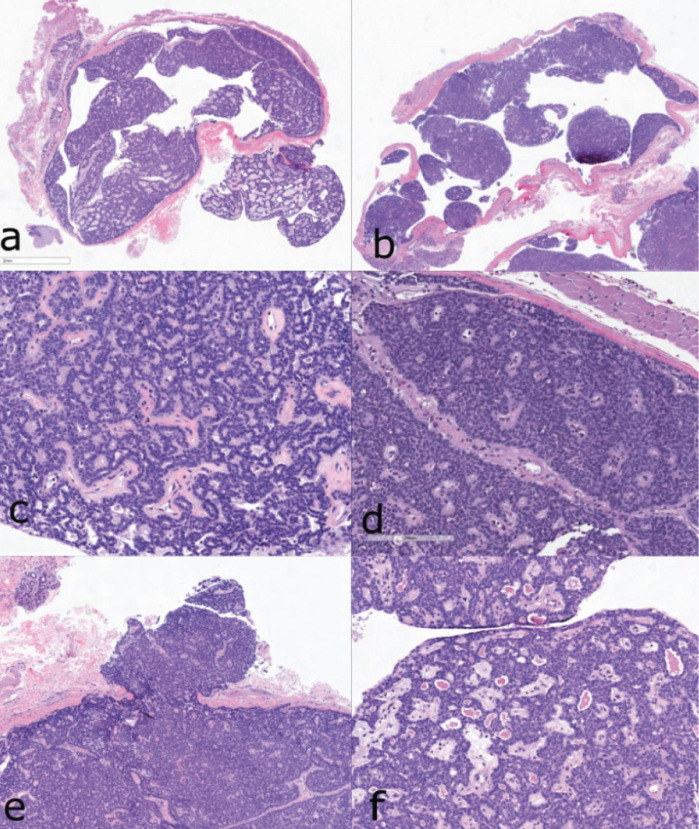
Patient 2. Histopathologic features of the tumors involving the right and left buccal mucosa. Well-circumscribed and encapsulated epithelial proliferations with prominent central cystic degenerative changes in the left (a) and right (b) lesion. Cuboidal or columnar neoplastic cells forming anastomosing cords or beading structures (c) intermixed with areas of solid arrangement (d) within an inconspicuous stroma (c/right lesion and d/left lesion). e. Multinodular pattern with small cords of the right buccal tumor extending outside of the main mass. f. Tyrosine crystals within the luminal area of the ductal structures (left lesion). *Hematoxylin and eosin stain, initial magnification 200x*.

Histopathologic examination showed well-circumscribed and encapsulated epithelial proliferations by monomorphic cuboidal or columnar cells with deeply basophilic nuclei (Fig. 4a-d). The neoplastic cells formed single layered cords or exhibited characteristic beading architecture (Fig. 4c) within an inconspicuous stroma while focal areas of solid arrangement were observed (Fig. 4d). In both lesions, prominent cystic degenerative changes with a central cystic space lined by these epithelial proliferations were observed (Figs. 4a and 4b). Additionally, a multinodular pattern was noticed with small cords of the tumors extending outside of the main masses (Fig. 4e). Finally, eosinophilic depositions within the luminal component of ductal structures consistent with tyrosine crystals were obvious in the left nodule (Fig. 4f). Based on these histopathologic characteristics, a diagnosis of multifocal CA with prominent cystic morphology was rendered.

## Discussion

CA is considered one of the most noTable SGTs with predominance of cystic morphology (1,6). More precisely, this prominent tendency of forming cystic spaces has been supported by a recent extensive clinicopathologic study of 67 CA cases, of which this feature was observed in 62 (1). Occasional lesions with domination of cystic degeneration have been referred; however, sufficient emphasis about unicystic CAs has not been given.

After reviewing the English-language literature about histopathologically-proven unicystic CAs, a total number of 11 cases was recorded (3,4,7-10). Only lesions with histopathologic descriptions and/or photographic illustrations showing a single cystic cavity lined by the neoplastic proliferation were included while cases of CA showing multiple large cystic spaces were omitted. Unfortunately, in most of the reported cases, few data can be retrieved regarding their clinicopathologic features and biologic behavior, especially in comparison to conventional CA (4,8,9). The Table 1 presents the demographic data, clinical site, pathologic features and follow-up of predominantly cystic CAs, highlighting the aforementioned absence of sufficient data.

**Table 1:**
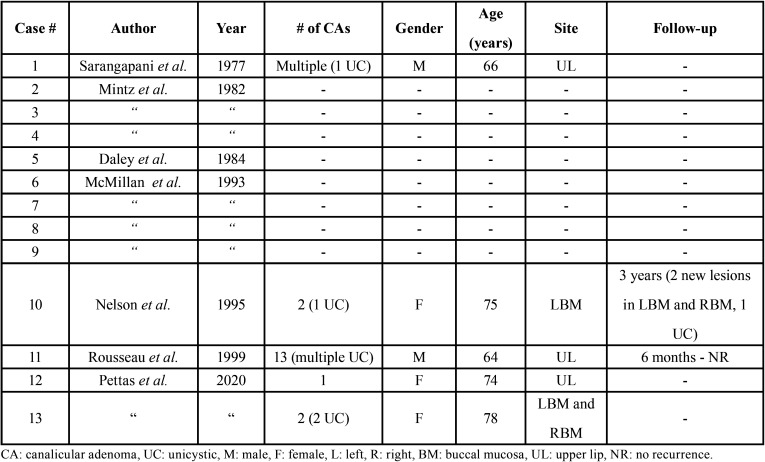
Cases of unicystic canalicular adenoma reported in the English-language literature.

Interestingly, in three patients (3,7,10) multiple CAs were reported, among which one or more exhibited a solitary cystic morphology. The first case of unicystic CA was reported in the context of multiple CAs of the upper lip in an adult male, in which one of the lesions presented with solitary cystic appearance (7). Subsequently, a 75 year-old female with two CAs of the left buccal mucosa (one with formation of a single cystic space) was reported (10). This specific patient developed metachronous lesions in the left and right buccal mucosa (one was also unicystic) (10). The patient with the highest number of simultaneous CAs described was a male with 13 lesions affecting the upper lip, with several of them exhibiting prominent cystic changes (3). Finally, in another study about monomorphic adenomas (8), four cases of unicystic CA as well as several multifocal tumors were described, however it was not specified whether these two different phenomena involved common patients.

Other reported multifocal CAs have also been described to exhibit a cystic appearance (11,12); however, the histopathologic description and microscopic illustrations in these articles did not particularly specify whether these tumors were unicystic, hence they were not included in our review. Even though the reported cases of unicystic CA are extremely few to draw the conclusion that this prominent cystic formation affects most commonly patients with multifocal tumors, further reporting of similar cases is encouraged.

To this date, many authors have tried to associate specific histopathologic features of SGTs to their cell of origin. Diverse theories have been proposed, including multi-cellular pattern of histogenesis implicating intercalated along with excretory duct reserve cells (13), while other reports imply the presence of a common stem cell undergoing specific genetic changes, leading to particular differentiation (14). Despite the fact that cystic salivary gland neoplasms have been extensively described, the association of this phenotypical characteristic with an underlying pattern of histogenesis has not been clarified. Cystic SGTs do not show predilection for a specific histogenetic profile, as tumors showing similarities to both 1) striated or excretory ducts (such as mucoepidermoid carcinoma and salivary duct carcinoma) and 2) acini or intercalated ducts (such as acinic cell carcinoma) (15) may be cystic. As a result, the complex and vague etiopathogenesis of CA [for which all different types of luminal cells have been implicated (1)] cannot be further explained by the commonly encountered cystic degeneration. However, a theory regarding CA morphogenesis underlines that separate genetic events are obvious in different phenotypic areas (16), which could suggest that prominently cystic tumors are molecularly distinct. Finally, the development of multiple CAs has not been elucidated neither with regards to the histogenetic origin of the tumor nor to the exact pathogenetic events mediated, with field changes as well as developmental defects being implicated for multifocality.

Besides the uncommon finding of solitary cystic morphology, other microscopic peculiarities that emphasize the heterogeneous pathologic phenotype of CA were noticed in both of our cases. More specifically, the first case did not exhibit conspicuous beading pattern (1) and the cytological features raised a differential diagnosis with BCA. However, the clinical presentation of a tumor affecting the upper lip of a 75 year-old female and the microscopic features of prominent cystic degeneration and no palisading of the peripheral neoplastic cells favored the diagnosis of CA. Immunohistochemical expression and positive staining localization is helpful to differentiate CA and BCA (1,17-20). More specifically, CK7 is expressed in the inner-epithelial cells, while SMA and p63 highlight the outer-myoepithelial component of BCAs (17). In contrast, CK7 is uniformely positive in CAs (1), SMA is negative (1), and p63 has been reported to be either negative (18) or cytoplasmically expressed and/or highlighting the squamous balls/morules (1). S-100 is diffusely positive in the tumor cells of CAs (1) and positive in the stromal component with variable immunoreactivity in the tumor cells of BCA (1,19). CD117 (c-kit) is positive in CAs (1) and most commonly negative or weakly positive in BCAs (17,20). All of these immunohistochemical findings were in accordance with the diagnosis of CA in our case while GFAP which is normally positive in the tumor - connective tissue interface of CAs was negative which could be attributed to the minimal amounts of stroma detected in the section (1). In the second case, besides the multifocal appearance as well as multilobular pattern within each tumor, tyrosine crystals were noticed which to our knowledge have been observed in only one case of the literature (1).

## Conclusions

CA is an uncommon SGT which exhibits characteristic clinicopathologic features in the majority of cases, but it occasionally diverges from the typical phenotype, causing diagnostic dilemmas. The prominent unicystic morphology, which has rarely been emphasized in the literature, can clinically mimic benign reactive processes. In addition, it highlights the complex histogenesis of this tumor and may raise significant debate about its biologic behavior. Moreover, in our first case, the absence of prominent beading pattern as well as the basaloid morphology of the tumor cells displayed similarities with BCA. The second case also exhibited uncommon features among which the most distinctive was the presence of multifocality. Despite the very low number of reported unicystic CAs, a tendency to occur in multifocal cases is worth noticed, raising questions regarding a possible association between unicystic (or predominantly cystic) morphology and multifocality.
